# Unifying aspect-based sentiment analysis BERT and multi-layered graph convolutional networks for comprehensive sentiment dissection

**DOI:** 10.1038/s41598-024-61886-7

**Published:** 2024-06-25

**Authors:** Kamran Aziz, Donghong Ji, Prasun Chakrabarti, Tulika Chakrabarti, Muhammad Shahid Iqbal, Rashid Abbasi

**Affiliations:** 1 Key Laboratory of Aerospace Information Security and Trusted Computing Ministry of Education, School of Cyber Science and Engineering, Wuhan University, Wuhan, China; 2 Department of Computer Science and Engineering, Sir Padampat Singhania University, Udaipur, 313601 Rajasthan India; 3 Department of Chemistry, Sir Padampat Singhania University, Udaipur, 313601 Rajasthan India; 4 Department of Computer science and Information Technology, Women university, Bagh, Azad Jammu and Kashmir Pakistan; 5 School of Computer Science and Artifical Intelligent, Wenzhou University, Wenzhou, 325035 China

**Keywords:** Data mining, Machine learning

## Abstract

Aspect-Based Sentiment Analysis (ABSA) represents a fine-grained approach to sentiment analysis, aiming to pinpoint and evaluate sentiments associated with specific aspects within a text. ABSA encompasses a set of sub-tasks that together facilitate a detailed understanding of the multifaceted sentiment expressions. These tasks include aspect and opinion terms extraction (ATE and OTE), classification of sentiment at the aspect level (ALSC), the coupling of aspect and opinion terms extraction (AOE and AOPE), and the challenging integration of these elements into sentiment triplets (ASTE). Our research introduces a comprehensive framework capable of addressing the entire gamut of ABSA sub-tasks. This framework leverages the contextual strengths of BERT for nuanced language comprehension and employs a biaffine attention mechanism for the precise delineation of word relationships. To address the relational complexity inherent in ABSA, we incorporate a Multi-Layered Enhanced Graph Convolutional Network (MLEGCN) that utilizes advanced linguistic features to refine the model’s interpretive capabilities. We also introduce a systematic refinement approach within MLEGCN to enhance word-pair representations, which leverages the implicit outcomes of aspect and opinion extractions to ascertain the compatibility of word pairs. We conduct extensive experiments on benchmark datasets, where our model significantly outperforms existing approaches. Our contributions establish a new paradigm for sentiment analysis, offering a robust tool for the nuanced extraction of sentiment information across diverse text corpora. This work is anticipated to have significant implications for the advancement of sentiment analysis technology, providing deeper insights into consumer preferences and opinions for a wide range of applications.

## Introduction

**Figure 1:**
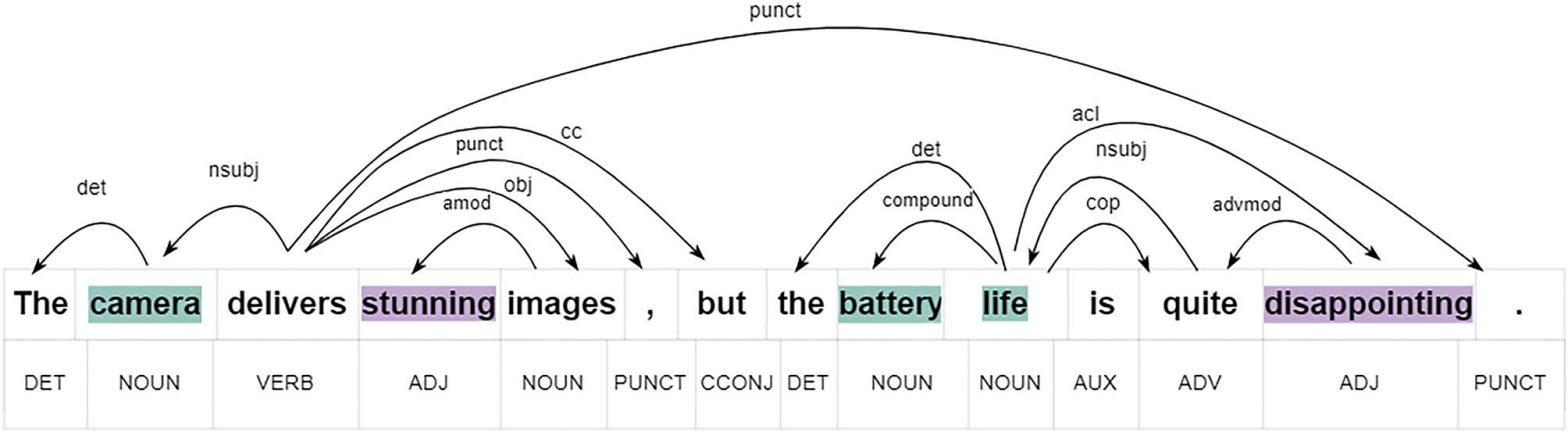
Universal dependency and part-of-speech tagging for the given example.

Aspect Based Sentiment Analysis represents a granular approach to parsing sentiments in text, focusing on the specific aspects or features discussed and the sentiment directed towards them^1–4^. It begins with ATE, which identifies the nouns or phrases that represent the focal points of sentiment within the text^5–7^. Then, OTE locates the adjectives or adverbs that express feelings or attitudes towards these aspects^8–10^. Moving beyond identification, ALSC categorizes the sentiment towards each aspect as positive, negative, or neutral^11–13^. Aspect-oriented Opinion Extraction then associates these sentiments with the corresponding aspects^14,15^, while Aspect Extraction and Sentiment Classification combines the processes of ATE and ALSC for efficiency^16^. Aspect-Opinion Pair Extraction is the process of pairing each aspect with its qualifying opinion, and the most integrative task, ASTE, combines aspects, opinions, and sentiments into a comprehensive triplet for each aspect mentioned^17–20^. The Figure 1 presents an example sentence annotated with universal dependency and part of speech, while Table 1 displays the outcomes of various subcomponents of sentiment analysis for this particular review.

tasks intricately mines text data to identify sentiments toward specific aspects mentioned within^21,22^. In evaluating a smartphone review like “The camera delivers stunning images, but the battery life is quite disappointing,” ABSA performs a series of sophisticated sub-tasks: ATE identifies the features “camera” and “battery life” under scrutiny; OTE captures the corresponding evaluative terms “stunning” and “disappointing”; Aspect-Level Sentiment Classification (ALSC) assigns sentiments, labeling the camera’s as positive and the battery’s as negative. AOE links these sentiments to their respective aspects, crafting a direct association between “stunning” and “camera” and between “disappointing” and “battery life”. Aspect Extraction and Sentiment Classification (AESC) streamlines the process by tagging “camera” with a positive sentiment and “battery life” with a negative sentiment in one step. AOPE then pairs aspects with their opinions, forming the pairs (“camera”, “stunning”) and (“battery life”, “disappointing”), which is critical for pinpointing precise consumer attitudes. Finally, Aspect Sentiment Triplet Extraction integrates these elements, producing a comprehensive sentiment overview with triplets (“camera”, “stunning”, positive) and (“battery life”, “disappointing”, negative), offering granular insights into the multifaceted nature of consumer feedback^22–26^.

**Table 1:** ABSA sub-tasks and their outputs for a given example.

Sub-task	Output
AT	{camera, battery life}
OT	{stunning, disappointing}
ALSC	{(camera, Positive), (battery life, Negative)}
AOE	{(camera, stunning), (battery life, disappointing)}
AESC	{(camera, Positive), (battery life, Negative)}
AOPE	{(camera, stunning), (battery life, disappointing)}
ASTE	{(camera, stunning, Positive),
	(battery life, disappointing, Negative)}

The implementation of ABSA is fraught with challenges that stem from the complexity and nuances of human language^27,28^. One significant hurdle is the inherent ambiguity in sentiment expression, where the same term can convey different sentiments in different contexts. Moreover, sarcasm and irony pose additional difficulties, as they often invert the literal sentiment of terms, requiring sophisticated detection techniques to interpret correctly^29^. Another challenge is co-reference resolution, where pronouns and other referring expressions must be accurately linked to the correct aspects to maintain sentiment coherence^30,31^. Additionally, the detection of implicit aspects, where sentiments are expressed without explicitly mentioning the aspect, necessitates a deep understanding of implied meanings within the text. Furthermore, multilingual and cross-domain ABSA require models that can transfer knowledge and adapt to various languages and domains, given that sentiment indicators and aspect expressions can vary significantly across cultural and topical boundaries^32–35^. The continuous evolution of language, especially with the advent of internet slang and new lexicons in online communication, calls for adaptive models that can learn and evolve with language use over time. These challenges necessitate ongoing research and development of more sophisticated ABSA models that can navigate the intricacies of sentiment analysis with greater accuracy and contextual sensitivity.

To effectively navigate the complex landscape of ABSA, the field has increasingly relied on the advanced capabilities of deep learning. Neural sequential models like Long Short-Term Memory (LSTM) and Gated Recurrent Units (GRU) have set the stage by adeptly capturing the semantics of textual reviews^36–38^. These models contextualize the sequence of words, identifying the sentiment-bearing elements within. The Transformer architecture, with its innovative self-attention mechanisms, along with Embeddings from Language Models (ELMo), has further refined the semantic interpretation of texts^39–41^. These advancements have provided richer, more nuanced semantic insights that significantly enhance sentiment analysis. However, despite these advancements, challenges arise when dealing with the complex syntactic relationships inherent in language-connections between aspect terms, opinion expressions, and sentiment polarities^42–44^. To bridge this gap, Tree hierarchy models like Tree LSTM and Graph Convolutional Networks (GCN) have emerged, integrating syntactic tree structures into their learning frameworks^45,46^. This incorporation has led to a more granular analysis that combines semantic depth with syntactic precision, allowing for a more accurate sentiment interpretation in complex sentence constructions. Furthermore, the integration of external syntactic knowledge into these models has shown to add another layer of understanding, enhancing the models’ performance and leading to a more sophisticated sentiment analysis process.

In our approach to ABSA, we introduce an advanced model that incorporates a biaffine attention mechanism to determine the relationship probabilities among words within sentences. This mechanism generates a multi-dimensional vector where each dimension corresponds to a specific type of relationship, effectively forming a relation adjacency tensor for the sentence. To accurately capture the intricate connections within the text, our model converts sentences into a multi-channel graph. This graph treats words as nodes and the elements of the relation adjacency tensor as edges, thereby mapping the complex network of word relationships. Our model stands out by integrating a wide array of linguistic features. These include lexical and syntactic information such as part-of-speech tags, types of syntactic dependencies, tree-based distances, and relative positions between pairs of words. Each set of features is transformed into edges within the multi-channel graph, substantially enriching the model’s linguistic comprehension. This comprehensive integration of linguistic features is novel in the context of the ABSA task, particularly in the ASTE task, where such an approach has seldom been applied. Additionally, we implement a refining strategy that utilizes the outcomes of aspect and opinion extractions to enhance the representation of word pairs. This strategy allows for a more precise determination of whether word pairs correspond to aspect-opinion relationships within the context of the sentence. Overall, our model is adept at navigating all seven sub-tasks of ABSA, showcasing its versatility and depth in understanding and analyzing sentiment at a granular level.

- We present an advanced neural network architecture that addresses every sub-task associated with Aspect-Based Sentiment Analysis (ABSA). This model establishes a new benchmark for the integration of syntactic and semantic data. It markedly improves the accuracy in detecting aspect and opinion terms along with their corresponding sentiment classifications.
- Our research integrates an extensive range of linguistic features, such as syntactic dependencies and part-of-speech patterns, into the ABSA framework. This integration substantially enhances the model’s ability to capture the nuances of language, leading to improved sentiment analysis accuracy.
- We have crafted a novel refining strategy that utilizes the initial results of aspect and opinion extractions. This strategy refines the representation of word pairs, sharpening the alignment between aspects and their corresponding opinions. This step is vital for the precise detection of sentiment orientations and intensities, which lie at the heart of ABSA.

The remainder of this paper is organized as follows: In Sect. Related work, we discuss the relevant literature and prior work in the domain. Section Proposed framework delves into the proposed framework for the model our proposed methodology, encompassing the techniques and algorithms we employed, Sect. Experiments showcases the experimental results, and the evaluation is presented in Sect. Model analysis we perform an ablation study. Finally, Sect. Conclusion concludes the paper, summarizing our contributions and suggesting potential avenues for future research.

## Related work

In this segment, we explore the landscape of Aspect Based Sentiment Analysis research, focusing on both individual tasks and integrated sub-tasks. We begin by delving into early research that highlights the application of graph neural network models in ABSA. This is followed by an examination of studies that leverage attention mechanisms and pre-trained language models, showcasing their impact and evolution in the field of ABSA.

### Aspect based sentiment analysis and its subtasks

The field of ABSA has garnered significant attention over the past ten years, paralleling the rise of e-commerce platforms. Xue and Li present a streamlined convolutional neural network model with gating mechanisms for ABSA, offering improved accuracy and efficiency over traditional LSTM and attention-based methods, particularly in aspect-category and aspect-term sentiment analysis^47^. Ma et al. enhance ABSA by integrating commonsense knowledge into an LSTM with a hierarchical attention mechanism, leading to a novel ’Sentic LSTM’ that outperforms existing models in targeted sentiment tasks^48^. Yu et al. propose a multi-task learning framework, the Multiplex Interaction Network (MIN), for ABSA, emphasizing the importance of ATE and OTE. Their approach, which adeptly handles interactions among subtasks, showcases flexibility and robustness, especially in scenarios where certain subtasks are missing, and their model’s proficiency in both ATE and OTE stands out in extensive benchmark testing^49^. Dai et al. demonstrate that fine-tuned RoBERTa (FT-RoBERTa) models, with their intrinsic understanding of sentiment-word relationships, can enhance ABSA and achieve state-of-the-art results across multiple languages^50^. Chen et al. propose a Hierarchical Interactive Network (HI-ASA) for joint aspect-sentiment analysis, which excels in capturing the interplay between aspect extraction and sentiment classification. This method, integrating a cross-stitch mechanism for feature blending and mutual information for output constraint, showcases the effectiveness of interactive tasks, particularly in Aspect Extraction and Sentiment Classification (AESC)^51^. Zhao et al. address the challenge of extracting aspect-opinion pairs in ABSA by introducing an end-to-end Pair-wise Aspect and Opinion Terms Extraction (PAOTE) method. This approach diverges from traditional sequence tagging by considering the task through the lens of joint term and relation extraction, utilizing a multi-task learning framework that supervises term extraction via span boundaries while concurrently identifying pair-wise relations. Their extensive testing indicates that this model sets a new benchmark, surpassing previous state-of-the-art methods^52,53^.

Innovations in ABSA have introduced models that outpace traditional methods in efficiency and accuracy. New techniques integrating commonsense knowledge into advanced LSTM frameworks have improved targeted sentiment analysis^54^. Multi-task learning models now effectively juggle multiple ABSA subtasks, showing resilience when certain data aspects are absent. Pre-trained models like RoBERTa have been adapted to better capture sentiment-related syntactic nuances across languages. Interactive networks bridge aspect extraction with sentiment classification, offering more complex sentiment insights. Additionally, novel end-to-end methods for pairing aspect and opinion terms have moved beyond sequence tagging to refine ABSA further. These strides are streamlining sentiment analysis and deepening our comprehension of sentiment expression in text^55–59^.

### Innovative approaches to sentiment analysis leveraging attention mechanisms

Attention mechanisms have gained traction in deep learning(DL) models addressing ABSA sub-components, recognized for their effectiveness in semantically linking aspects with contextual words. In addressing aspect-based sentiment classification, Liu et al. identified a gap in current neural attention models, which tend to highlight sentiment words without adequately linking them to the relevant aspects within a sentence. This shortcoming becomes particularly evident in sentences with multiple aspects and complex structures. They introduced a novel attention-based model that incorporates dual mechanisms: a sentence-level attention for global aspect relevance, and a context-level attention that accounts for the sequence and interrelation of words. Their empirical results showed that this dual mechanism approach significantly improves performance over existing models^60^. Lin et al. advanced the interpretability of sentence embeddings by leveraging a self-attention mechanism. Their novel approach represents embeddings as 2-D matrices, allowing each row to focus on distinct segments of a sentence. This not only enhances the model’s performance on tasks such as author profiling, sentiment classification, and textual entailment, but also provides an intuitive method for visualizing the parts of the sentence that contribute to the embedding’s formation^61^.Chen et al. explored the integration of Graph Convolutional Networks (GCN) with co-attention mechanisms to enhance aspect-based sentiment analysis (ABSA). Their model effectively utilizes both semantic and syntactic information to filter out irrelevant context, demonstrating significant improvements in identifying the sentiment polarity of specific aspects within sentences^62^. Wang et al. targeted the challenge of discerning sentiment polarity towards specific aspects in text, a task complicated by the subtleties of language and the presence of multiple aspects within a single sentence. Their solution involves a novel encoding of syntactic information into a unified aspect-oriented dependency tree structure. By deploying a relational graph attention network (R-GAT) that operates on this refined tree structure, their method more accurately identifies connections between aspects and opinion words, leading to notable improvements in sentiment analysis performance on prominent datasets^63^.

Attention mechanisms have revolutionized ABSA, enabling models to home in on text segments critical for discerning sentiment toward specific aspects^64^. These models excel in complex sentences with multiple aspects, adjusting focus to relevant segments and improving sentiment predictions. Their interpretability and enhanced performance across various ABSA tasks underscore their significance in the field^65–67^.

### Syntax-driven approaches to aspect-level sentiment analysis

Zhang and Qian’s model improves aspect-level sentiment analysis by using hierarchical syntactic and lexical graphs to capture word co-occurrences and differentiate dependency types, outperforming existing methods on benchmarks^68^. In the field of ALSC, Zheng et al. have highlighted the importance of syntactic structures for understanding sentiments related to specific aspects. Their novel neural network model, RepWalk, leverages replicated random walks on syntax graphs to better capture the informative contextual words crucial for sentiment analysis. This method has shown superior performance over existing models on multiple benchmark datasets, underscoring the value of incorporating syntactic structure into sentiment classification representations^69^. Zhang and Li’s research advances aspect-level sentiment classification by introducing a proximity-weighted convolution network that captures syntactic relationships between aspects and context words. Their model enhances LSTM-derived contexts with syntax-aware weights, effectively distinguishing sentiment for multiple aspects and improving the overall accuracy of sentiment predictions^70^. Huang and Li’s work enhances aspect-level sentiment classification by integrating syntactic structure and pre-trained language model knowledge. Employing a graph attention network on dependency trees alongside BERT’s subword features, their approach achieves refined context-aspect interactions, leading to more precise sentiment polarity determinations in complex sentences^71^. Xu, Pang, Wu, Cai, and Peng’s research focuses on leveraging comprehensive syntactic structures to improve aspect-level sentiment analysis. They introduce “Scope” as a novel concept to outline structural text regions pertinent to specific targets. Their hybrid graph convolutional network (HGCN) merges insights from both constituency and dependency tree analyses, enhancing sentiment-relation modeling and effectively sifting through noisy opinion words^72^. Xiao et al. enhance aspect-based sentiment classification by introducing a graph neural network model that leverages a part-of-speech guided syntactic dependency graph and a syntactic distance attention layer, significantly outperforming traditional methods on public datasets^73^. Incorporating syntax-aware techniques, the Enhanced Multi-Channel Graph Convolutional Network (EMC-GCN) for ASTE stands out by effectively leveraging word relational graphs and syntactic structures. Its use of biaffine attention to construct relation-aware representations, combined with a unique refining strategy for syntactically-informed word-pair representations, results in significant improvements over existing methods as evidenced by benchmark dataset performances^19^.

The integration of syntactic structures into ABSA has significantly improved the precision of sentiment attribution to relevant aspects in complex sentences^74,75^. Syntax-aware models excel in handling sentences with multiple aspects, leveraging grammatical relationships to enhance sentiment discernment. These models not only deliver superior performance but also offer better interpretability, making them invaluable for applications requiring clear rationale. The adoption of syntax in ABSA underscores the progression toward more human-like language processing in artificial intelligence^76–78^.

While existing literature lays a solid groundwork for Aspect-Based Sentiment Analysis, our model addresses critical limitations by advancing detection and classification capabilities in complex linguistic contexts. Our Multi-Layered Enhanced Graph Convolutional Network (MLEGCN) integrates a biaffine attention mechanism and a sophisticated graph-based approach to enhance nuanced text interpretation. This model effectively handles multiple sentiments within a single context and dynamically adapts to various ABSA sub-tasks, improving both theoretical and practical applications of sentiment analysis. This not only overcomes the simplifications seen in prior models but also broadens ABSA’s applicability to diverse real-world datasets, setting new standards for accuracy and adaptability in the field.

## Proposed framework

In this section, we introduce the formal definitions pertinent to the sub-tasks of ABSA. Figure 3 is the overall architecture for Fine-grained Sentiments Comprehensive Model for Aspect-Based Analysis. Following these definitions, we then formally outline the problem based on these established terms.

Given an input sentence $S = \{w_1, w_2, \ldots , w_n\}$ comprising $n$ words, our model excels in performing seven subtasks of ABSA. It identifies $a$ as an aspect term and $o$ as an opinion term, while $s$ represents the sentiment polarity associated with the aspect. This sentiment polarity is classified within a label set $X = \{\text {POS}, \text {NEU}, \text {NEG}\}$, encompassing three sentiment polarities: positive, neutral, and negative. The model processes the sentence to discern and interpret these specific elements.

1. Aspect Term Extraction (ATE): Extracts all aspect terms from the given sentence $S$.
  ATE: $$A = \{a_i | a_i \in S\}$$
2. Opinion Term Extraction (OTE): Identifies all opinion terms within the sentence $S$.
  OTE: $$O = \{o_j | o_j \in S\}$$
3. Aspect Level Sentiment Classification (ALSC): Predicts the sentiment polarity of each aspect term in $S$, with polarities defined in $X$.
  ALSC: $$S_A = \{s(a_i) | a_i \in A, s(a_i) \in X\}$$
4. Aspect-Opinion Pair Extraction (AOE): Extracts pairs of aspect terms and their corresponding opinion terms from $S$.
  AOE: $$AO = \{(a_i, o_j) | a_i \in A, o_j \in O\}$$
5. Aspect and Sentiment Co-Extraction (AESC): Simultaneously identifies aspect terms and their sentiments from $S$.
  AESC: $$AS = \{(a_i, s(a_i)) | a_i \in A, s(a_i) \in X\}$$
6. Aspect-Opinion Pairing (AOP): Finds pairs of aspect and opinion terms that are related within $S$.
  AOP: $$AOM = \{(a_i, o_j) | a_i \in A, o_j \in O, \text {related}\}$$
7. Aspect-Sentiment-Triplet Extraction (ASTE): Forms triplets from $S$ that consist of an aspect term, opinion term, and sentiment polarity.
  ASTE: $$T = \{(a_i, o_j, s_k) | a_i \in A, o_j \in O, s_k \in X\}$$

### Relation definition and table filling

**Figure 2:**
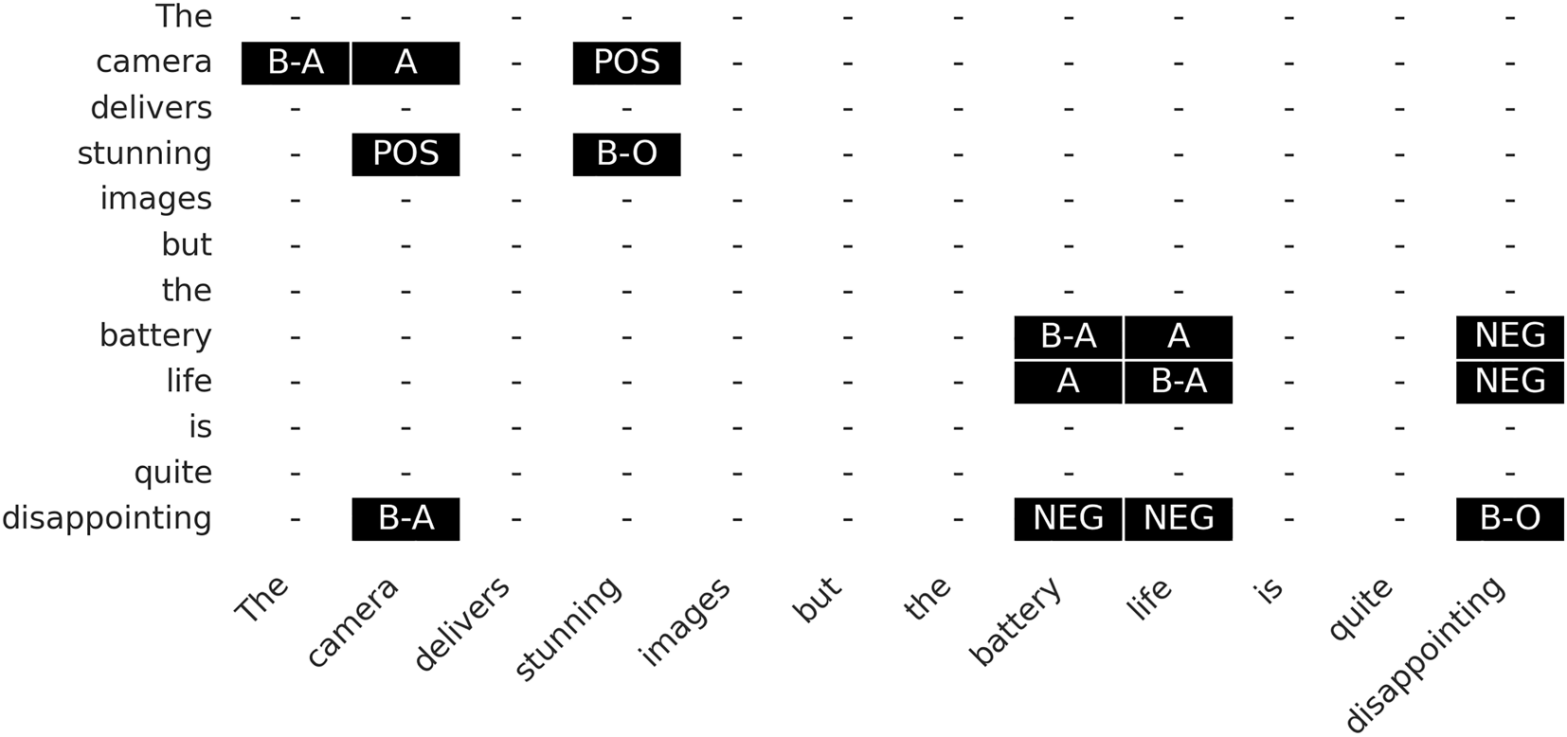
Table filling for ABSA in a sentence is illustrated. Each cell denotes a word pair with a relation or label.

**Figure 3:**
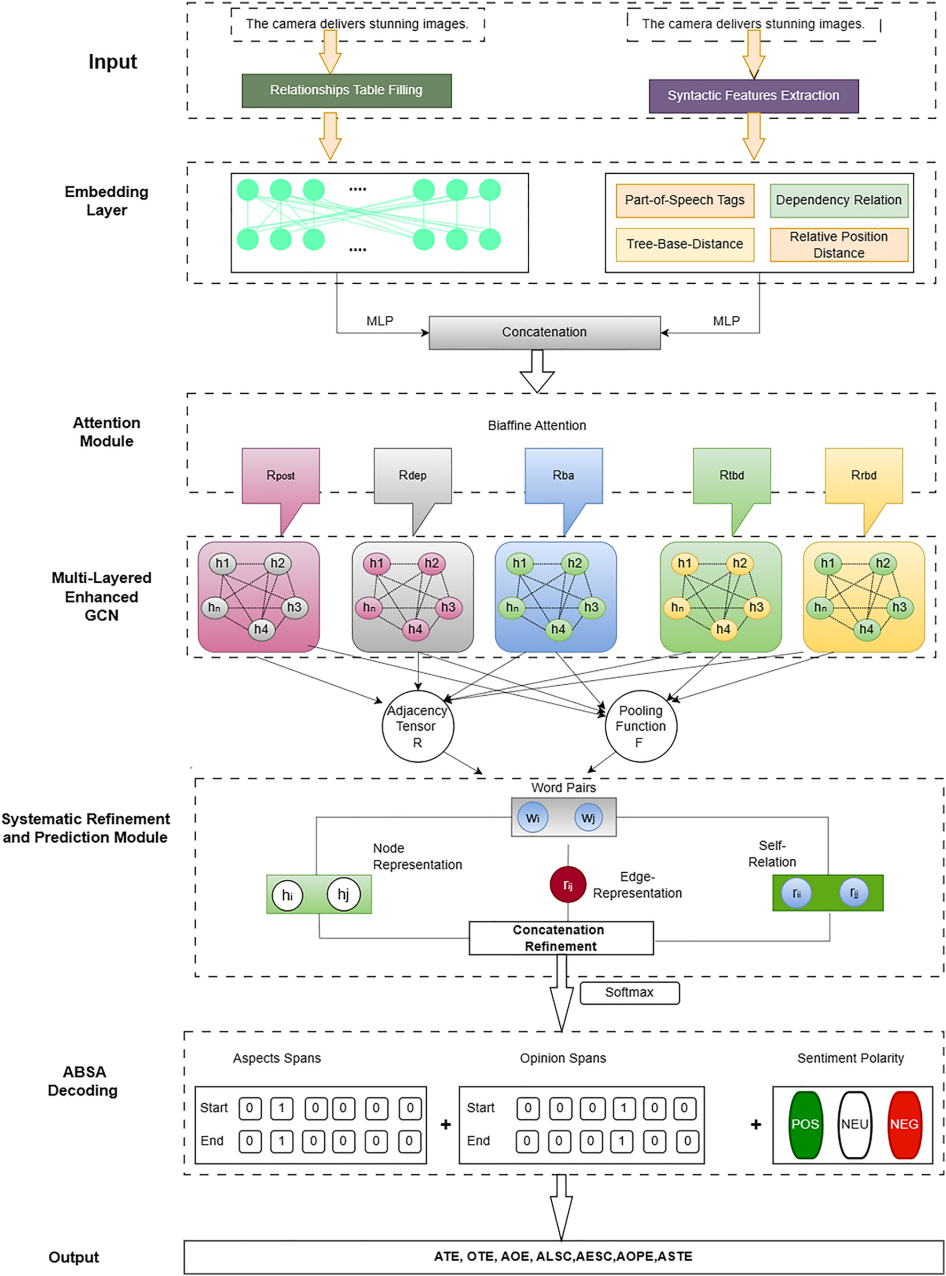
The overall architecture fine-grained sentiments comprehensive model for aspect-based analysis.

The study employs a framework that categorizes word relationships within sentences into ten distinct types, following the methodology introduced by Chen et al.^19^. Four specific labels—{B-A, I-A, B-O, I-O}—are applied to accurately identify terms that represent aspects and opinions. This refined strategy enhances boundary definition within the model, offering improvements over the GTS approach previously outlined by Wu et al^79^. The ‘B’ and ‘I’ labels signify the start and continuation of a term, respectively. Additionally, the suffixes -A and -O are used to categorize a term as either an aspect or an opinion. In Table 1, the A and O relations assist in determining whether pairs of distinct words pertain to the same aspect or opinion term. Moreover, the sentiment relations—{POS, NEU, NEG}—serve a dual purpose: they confirm whether word pairs correspond and also ascertain the sentiment polarity linked with aspect-opinion pairs. By implementing the table-filling technique, as detailed by Miwa & Sasaki^80^ and Gupta et al.^81^, a relation table is constructed for each sentence with annotations. This process is exemplified in Figure 2, which illustrates the word pairs along with their designated relations, with each table cell denoting a specific word-to-word relationship (Figure 3).

**Algorithm 1:**
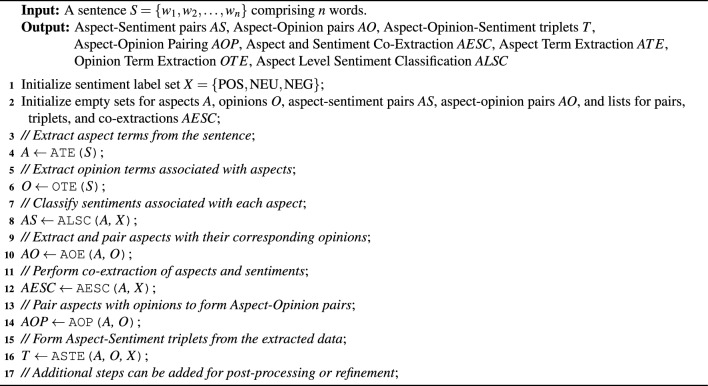
Comprehensive ABSA Algorithm with Classified Subtasks.

### Model layers and formation

#### Input layer

BERT, short for *Bidirectional Encoder Representations from Transformers*, was introduced by Devlin et al. in 2019. This model has been widely recognized for its outstanding performance on various natural language processing tasks. BERT utilizes a deep learning technique known as the Transformer, which employs attention mechanisms to capture contextual information from all words in a sentence, irrespective of their positions^82^. When BERT processes an input sentence $S$, which consists of a sequence of tokens $S = \{w_1, w_2, \ldots , w_n\}$ where $n$ is the number of tokens, it generates a corresponding sequence of hidden states $H = \{h_1, h_2, \ldots , h_n\}$. These hidden states are derived from the last layer of the Transformer block within BERT, capturing the nuanced contextual relationships between the input tokens. This representation power of BERT enables it to serve as an effective sentence encoder for various downstream tasks, providing enriched feature representations that can significantly enhance the performance of natural language understanding systems.

#### Attention module

In our model, we employ a biaffine attention module to determine the relational probability distribution between word pairs in a sentence. The effectiveness of biaffine attention in syntactic dependency parsing is well-documented^83^. The biaffine attention mechanism is defined by several key equations, as outlined below:

- Equation (1) defines the transformation of hidden state $h_i$ through the attention module:
  1 $$\begin{aligned} h^a_i = \text {MLP}_a(h_i) \end{aligned}$$
- Equation (2) similarly transforms hidden state $h_j$:
  2 $$\begin{aligned} h^o_j = \text {MLP}_o(h_j) \end{aligned}$$
- Equation (3) calculates the interaction score $g_{i,j}$ for word pairs:
  3 $$\begin{aligned} g_{i,j} = h^a_i {}^T U^1 h^o_j + U^2 (h^a_i \oplus h^o_j) + b \end{aligned}$$
- Equation (4) normalizes these scores to determine the relation probability $r_{i,j,k}$ for each relation type:
  4 $$\begin{aligned} r_{i,j,k} = \frac{\exp (g_{i,j,k})}{\sum _{l=1}^m \exp (g_{i,j,l})} \end{aligned}$$
- Finally, Equation (5) applies the biaffine attention to obtain the adjacency tensor $R$:
  5 $$\begin{aligned} R = \text {Biaffine}(\text {MLP}_a(H), \text {MLP}_o(H)) \end{aligned}$$

These equations collectively model the relations between words in a sentence, where $m$ represents the number of relation types, and each relation type corresponds to a channel in the adjacency tensor $R$. The trainable parameters $U^1$, $U^2$, and $b$, along with the concatenation operation $\oplus$, are integral to this process.

#### Multi-layered enhanced graph convolutional network (MLEGCN)

The MLEGCN represents a significant development over traditional Graph Convolutional Networks (GCN), designed to process graph-structured data more effectively in natural language processing tasks. Originating from the adaptation of Convolutional Neural Networks (CNNs) to graph data^84,85^, the MLEGCN enhances this model by introducing mechanisms that capture complex relational dynamics within sentences.

In the MLEGCN framework, each node in the graph corresponds to a word, while edges reflect the syntactic dependencies between these words. This setup facilitates in-depth modeling of sentence structures. The connections between nodes are represented using an adjacency matrix $A \in \mathbb {R}^{n \times n}$, where $n$ is the number of words in a sentence. In this matrix, $A_{ij} = 1$ indicates a direct syntactic link between the words corresponding to nodes $i$ and $j$, and $A_{ij} = 0$ otherwise.

A significant enhancement in MLEGCN is the integration of soft edges, which express the probabilistic strengths of connections between node pairs. This concept is inspired by advancements in attention mechanisms^86^, allowing the network to adjust the influence of each connection dynamically. The model incorporates a multi-channel adjacency tensor $R^{ba} \in \mathbb {R}^{n \times n \times m}$, where each channel $m$ corresponds to a unique type of relational dynamic, modulated through a biaffine attention module.

The computational operations in MLEGCN are detailed as follows:

6 $$\begin{aligned} H^{ba}_{k} = \sigma (R^{ba}_{:,:,k} H W_k + b_k) \quad \end{aligned}$$

7 $$\begin{aligned} {\hat{H}}^{ba} = f(H^{ba}_1, H^{ba}_2, \ldots , H^{ba}_m) \quad \end{aligned}$$

In Equation 6, $R^{ba}_{:,:,k}$ represents the $k$-th relational channel within $R^{ba}$. $W_k$ and $b_k$ denote the weight and bias specific to that channel. The function $\sigma$ is an activation function, such as ReLU, used to introduce non-linearity into the network. The function $f(\cdot )$, a pooling operation, combines the hidden representations from all channels to produce a unified node representation.

Through its channel-specific convolutions, MLEGCN is able to differentiate and analyze various types of word relationships. This capability allows for a more nuanced understanding of language, making MLEGCN particularly effective for tasks like sentiment analysis, entity recognition, and syntactic parsing. The consolidated output ${\hat{H}}^{ba}$, derived by pooling across channels (Equation 7), provides a holistic view of the word relationships, crucial for performing complex downstream tasks.

#### Enhanced understanding of syntactic features

Chen et al. 2022’s innovative framework employs a comprehensive suite of linguistic features that critically examine the interrelations between word pairs within sentences. These features, which include combinations of part-of-speech tags, varieties of syntactic dependencies, tree-based hierarchical distances, and relative positioning within the sentence, contribute to the detailed understanding of language structure.

In practical terms, the model initiates an examination of each word pair $(w_i, w_j)$ by assigning a self-dependency feature that signifies the inherent syntactic role associated with the words. This is operationalized through the initialization of four adjacency tensors: $R_{\text {psc}}$ for part-of-speech combinations, $R_{\text {dep}}$ for syntactic dependencies, $R_{\text {tbd}}$ for tree-based distances, and $R_{\text {rpd}}$ for relative positions-each offering a different perspective on the sentence structure.

Focusing on the syntactic dependency dimension as an illustrative case, when there is a recognized dependency type such as ’nsubj’ (nominal subject) between $w_i$ and $w_j$, the corresponding location in the tensor $R_{\text {dep}}$ is embedded with a vector representation of ’nsubj’. This embedding is retrieved from a dynamically learned table, encapsulating the relationship’s essence. Conversely, the absence of a dependency connection is indicated by a zero vector at the respective tensor indices.

The tensors undergo a process of graph convolutions, refining the raw node representations into enriched forms ${\hat{H}}_{\text {psc}}, {\hat{H}}_{\text {dep}}, {\hat{H}}_{\text {tbd}},$ and ${\hat{H}}_{\text {rpd}}$. Through techniques such as average pooling and concatenation, these representations are synthesized into holistic node and edge descriptors for the sentence as given by:

8 $$\begin{aligned} H = f({\hat{H}}_{\text {ba}}, {\hat{H}}_{\text {psc}}, {\hat{H}}_{\text {dep}}, {\hat{H}}_{\text {tbd}}, {\hat{H}}_{\text {rpd}}) \end{aligned}$$

9 $$\begin{aligned} R = R_{\text {ba}} \oplus R_{\text {psc}} \oplus R_{\text {dep}} \oplus R_{\text {tbd}} \oplus R_{\text {rpd}} \end{aligned}$$

Here, $H$ encapsulates the ensemble of node representations $\{h_1, h_2, \ldots , h_n\}$, while $R$ aggregates the edge representations $\{r_{1,1}, r_{1,2}, \ldots , r_{n,n}\}$ which collectively enhance the model’s proficiency in recognizing and interpreting complex linguistic constructs, thereby substantially improving its applicability in diverse NLP tasks.

**Figure 4:**
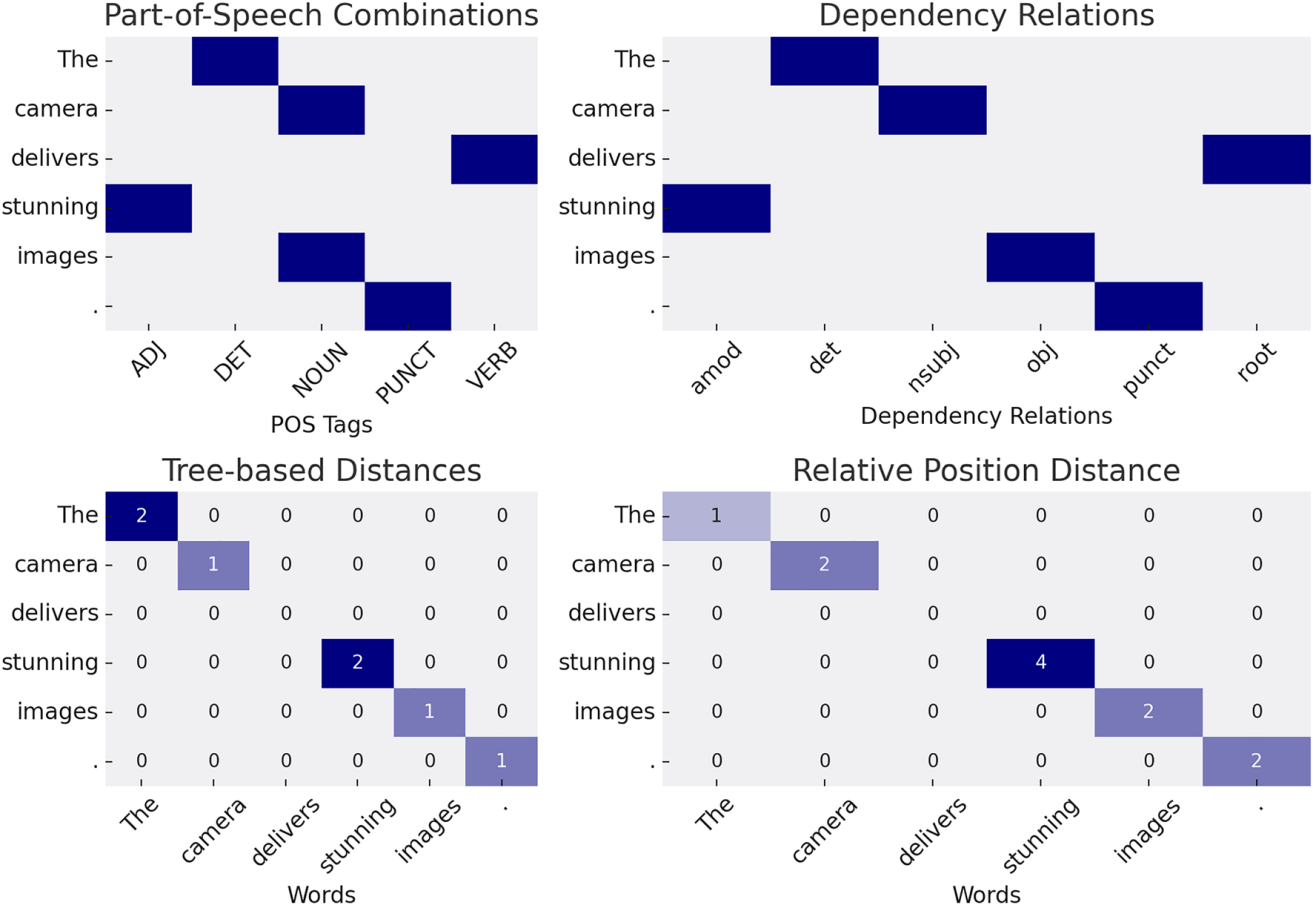
Matrices depicting the syntactic features leveraged by the framework for analyzing word pair relationships in a sentence, illustrating part-of-speech combinations, dependency relations, tree-based distances, and relative positions.

Figure 4 illustrates the matrices corresponding to the syntactic features utilized by the model. The Part-of-Speech Combinations and Dependency Relations matrices reveal the frequency and types of grammatical constructs present in a sample sentence. Similarly, the Tree-based Distances and Relative Position Distance matrices display numerical representations of word proximities and their respective hierarchical connections within the same sentence. These visualizations underscore the framework’s capacity to capture and quantify the syntactic essence of language.

#### Correlation constraints

To ensure the precise delineation of word relationships within a sentence, the model enforces a constraint on the adjacency tensor, which originates from the biaffine attention framework. This constraint is quantified by the following expression in equation 10:

10 $$\begin{aligned} L_{ba} = -\sum _{i=1}^{n} \sum _{j=1}^{n} \sum _{c \in C} I(y_{ij} = c) \log (r_{ij|c}) \end{aligned}$$

Where in the formula:

- $I(\cdot )$ stands for the indicator function.
- $y_{ij}$ denotes the verified relationship type between the word pair $(w_i, w_j)$.
- $C$ represents the comprehensive set of all potential relationship types.
- $r_{ij|c}$ is the model’s forecasted probability score for the relationship type $c$ between the word pair $(w_i, w_j)$.

In addition, this relational constraint is similarly applied to four other adjacency tensors, each linked to distinct linguistic features. These tensors are labeled as $L_{psc}$, $L_{dep}$, $L_{tbd}$, and $L_{rpd}$, correlating with individual linguistic feature sets.

#### Systematic refinement and prediction module

The predictive capabilities of our model are heavily reliant on accurately determining the sentiment relationship between word pairs $(w_i, w_j)$. This process begins with the combination of individual node representations $h_i$ and $h_j$, along with the edge representation $r_{ij}$, as illustrated in equation 11^87^. To enhance this initial representation, we introduce a systematic refinement strategy that utilizes additional self-referential edge representations $r_{ii}$ and $r_{jj}$. These are crucial in contexts where words may have self-related sentiment implications that affect their interaction with other words in the sentence.

**Refinement Strategy Rationale and Mechanism:**

- *Enhanced Contextual Understanding:* The inclusion of $r_{ii}$ and $r_{jj}$ allows our model to incorporate not only direct relational dynamics between $w_i$ and $w_j$ but also each word’s relationship with itself. This dual consideration is critical, especially in complex sentences where aspects and opinions can be nuanced.
- *Aspect and Opinion Extraction Influences:* When $w_i$ is an aspect and $w_j$ an opinion, the combined representation $s_{ij}$ is enriched by this systematic refinement approach. We leverage outcomes from aspect and opinion extractions to better assess and predict the potential sentiment (positive, neutral, or negative) based on empirical observations that aspects and opinions typically generate strong sentiment indicators.

**Figure 5:**
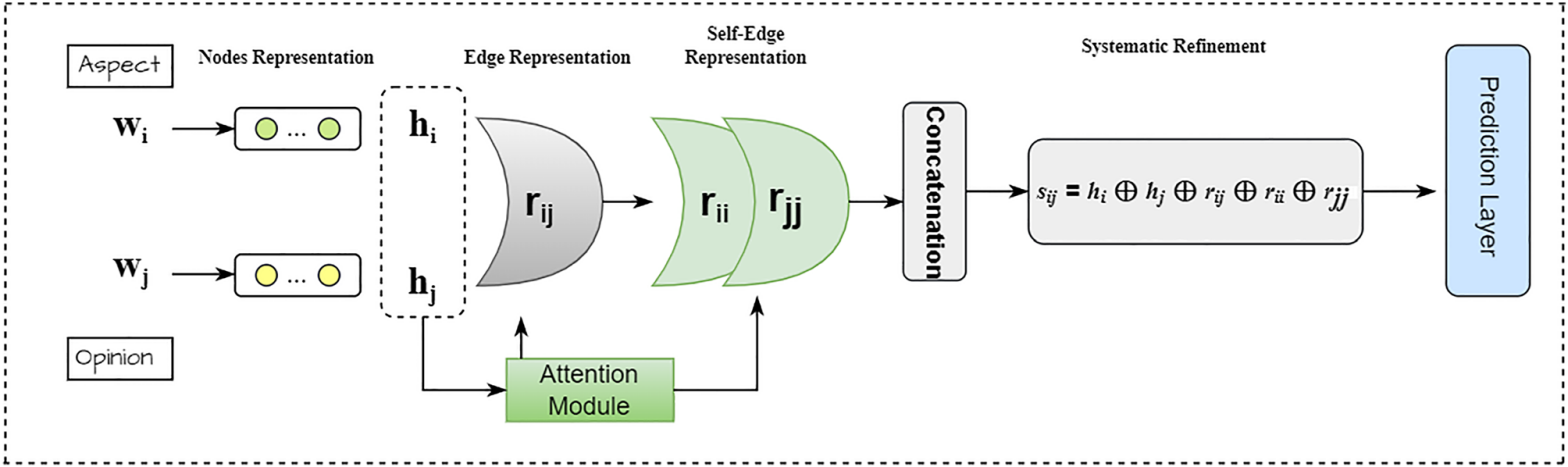
Illustration of systematic refinement.

11 $$\begin{aligned} s_{ij} = h_i \oplus h_j \oplus r_{ij} \oplus r_{ii} \oplus r_{jj} \end{aligned}$$

This refined representation $s_{ij}$ is processed through a linear layer followed by a softmax activation to calculate the probabilities of the sentiment label distribution also depicted in Figure 5:

12 $$\begin{aligned} p_{ij} = \text {softmax}(W_p s_{ij} + b_p) \end{aligned}$$

**Impact of Refinement on Prediction Accuracy:** To validate the effectiveness of our refinement strategy, we conducted error analysis comparing model outputs with and without the inclusion of self-referential edges. Our findings reveal that models incorporating $r_{ii}$ and $r_{jj}$ consistently perform better in scenarios involving implicit sentiment relations and complex aspect-opinion structures. Specifically, error rates decrease significantly in cases involving subtle sentiment expressions, underscoring the importance of our systematic refinement strategy. Equation 11 refines the representation of word pairs by integrating additional context that enhances the model’s sensitivity to nuanced linguistic features. Equation 12 then leverages this refined representation to predict the most likely sentiment label for each word pair, demonstrating a tangible improvement in the model’s ability to discern and classify sentiment relationships accurately. This enhancement is crucial for robust performance across diverse datasets and is supported by quantitative improvements in prediction accuracy in our experimental results section.

#### Loss function

The loss function we aim to minimize is given by:

13 $$\begin{aligned} L = L_p + \alpha L_{ba} + \beta (L_{psc} + L_{dep} + L_{tbd} + L_{rpd}) \end{aligned}$$

where:

- $L_p$ is the standard cross-entropy loss for the ASTE task, defined as:
  14 $$\begin{aligned} L_p = -\sum _{i=1}^n \sum _{j=1}^n \sum _{c \in C} I(y_{ij} = c) \log (p_{i,j|c}) \end{aligned}$$
- $\alpha$ and $\beta$ are coefficients that balance the influence of the different components of the loss function.
- $L_{ba}$, $L_{psc}$, $L_{dep}$, $L_{tbd}$, and $L_{rpd}$ represent additional loss components, addressing specific constraints and aspects of the task.

The structure of $L$ combines the primary task-specific loss with additional terms that incorporate constraints and auxiliary objectives, each weighted by their respective coefficients.

## Experiments

### Datasets

The study presents a detailed examination of a method’s efficacy when applied to two distinct benchmark datasets within the field of ABSA. These datasets are associated with the Semantic Evaluation (SemEval) challenges that occurred over the course of three consecutive years-2014 through 2016^88,89^.

The first of these datasets, referred to herein as Dataset 1 (D1), was introduced in a study by Wu et al. under the 2020a citation. The second dataset, known as Dataset 2 (D2), is the product of annotations by Xu et al. in 2020. It represents an enhanced and corrected version of an earlier dataset put forth by Peng et al. in 2020, aiming to rectify previous inaccuracies^79,90,91^.

Comprehensive metrics and statistical breakdowns of these two datasets are thoughtfully compiled in a section of the paper designated as Table 2. This table likely offers an in-depth look at the datasets, including the volume of data points, the assortment and balance of sentiment classifications, the variety of aspects evaluated, and other critical data that are essential for determining the strength and effectiveness of the ABSA methodology under review.

Additional resources and tools relevant to this study can be found at the following GitHub repositories: (https://github.com/xuuuluuu/SemEval-Triplet-data/tree/master/ASTE-Data-V2-EMNLP2020, https://github.com/huggingface/transformers, https://github.com/NJUNLP/GTS).

**Table 2:** Combined statistics for datasets D1 and D2.

	D1				D2			
	#S	#A	#O	#T	#S	#A	#O	#T
14res	1259	2064	2098	2356	1266	2051	2071	2338
	315	487	506	580	310	500	498	577
	493	851	861	1008	492	848	850	994
14lap	899	1257	1270	1452	906	1280	1264	1460
	225	332	313	383	219	295	304	346
	332	467	478	547	328	463	473	543
15res	603	871	966	1038	605	862	941	1013
	151	205	226	239	148	213	236	249
	325	436	469	493	322	432	461	485
16res	863	1213	1329	1421	857	1198	1307	1394
	216	298	331	348	210	296	319	339
	328	456	485	525	326	452	474	514

#### Implementation details

In our research, we have implemented the BERT-base-uncased version 5 as the core sentence encoder. To optimize this encoder, we employ the AdamW optimizer, as proposed by Loshchilov and Hutter (2018)^92^. This optimizer is specifically configured with a learning rate of $2 \times 10^{-5}$, a setting that is particularly tailored for fine-tuning the BERT component. For other trainable aspects of our model, a distinct learning rate of $10^{-3}$ is utilized. This bifurcation in learning rates is a strategic decision, ensuring that while the BERT model is fine-tuned with precision, other model components are trained more aggressively. Additionally, we set the dropout rate at 0.5 to mitigate the risk of overfitting, a common concern in deep learning models.

The architecture of our model is built with a keen eye on dimensionality, where the hidden state sizes for BERT and the Graph Convolutional Network (GCN) are set to 768 and 300, respectively. This difference reflects the varied complexity and nature of the data each component handles. Our model, termed MLEGCN, diverges from the traditional EMC-GCN framework. It undergoes an extensive training regime spanning 100 epochs, with each training batch comprising 16 samples. This epoch count and batch size are meticulously chosen to balance computational efficiency with effective learning. To manage the influence of relation constraints within our model, we meticulously tune two hyperparameters: $\alpha$ is set to 0.1 and $\beta$ to 0.01. This fine-tuning is crucial for balancing the relation dynamics in the model. It is noteworthy that the number of channels in our model is directly equivalent to the predefined number of relations, a design choice influenced by the immutable nature of these relation constraints.

For parsing and preparing the input sentences, we employ the Stanza tool, developed by Qi et al. (2020). Stanza is renowned for its robust parsing capabilities, which is critical for preparing the textual data for processing by our model. We ensure that the model parameters are saved based on the optimal performance observed in the development set, a practice aimed at maximizing the efficacy of the model in real-world applications^93^. Furthermore, to present a comprehensive and reliable analysis of our model’s performance, we average the results from five distinct runs, each initialized with a different random seed. This method provides a more holistic view of the model’s capabilities, accounting for variability and ensuring the robustness of the reported results.

#### Baselines

We evaluate the proposed method against a diverse set of baseline models, as detailed in Table 3. While many baseline models focus solely on specific subsets of the tasks associated with Aspect-Based Sentiment Analysis (ABSA), only a few provide comprehensive solutions for all associated sub-tasks.

- OTE-MTL^94^ conceptualizes ABSA as a process of extracting opinion triplets and utilizes a multi-task learning approach with distinct detection heads along with a sentiment dependency parser.
- Li-Unified+^95^ introduces a unified model for target-based sentiment analysis, employing dual RNNs to predict unified tags and determine target boundaries.
- RINANTE+^96^ uses rules derived from dependency parsing outputs to extract aspect and opinion terms, applying these rules on auxiliary data and refining the approach through a neural model.
- TS^97^ addresses the extraction of aspect sentiment triplets, advocating a two-step methodology for the prediction and association of aspects, opinions, and sentiments.
- CMLA+^98^ offers a comprehensive solution for the simultaneous extraction of aspect and opinion terms using a multi-layer attention mechanism.
- EMC-GCN^19^ incorporates word relationships within a multi-channel graph structure, representing these relationships as nodes and edges for extracting aspect sentiment triplets.
- SPAN-ASTE^99^ explores the interaction between complete spans of aspects and opinions to predict sentiment relationships essential for triplet extraction.
- IMN-BERT^100^ learns multiple tasks associated with ABSA at both token and document levels simultaneously, using a multi-task network approach.
- JET-BERT^101^ employs an end-to-end model for triplet extraction with a position-aware tagging scheme to capture complex interactions among triplets.
- DMRC^102^ tackles all ABSA tasks in a unified framework, jointly training two BERT MRC models with shared parameters.
- BMRC^103^ conceptualizes ASTE as a multi-turn MRC problem, deploying a bidirectional MRC architecture to identify sentiment triplets.
- BART-ABSA^104^ converts ABSA tasks into a generative model framework using BART for an integrated approach.
- SE-GCN^105^ presents a ’Syntax-Enhanced Graph Convolutional Network’, which integrates semantic and syntactic insights through graph convolution and attention mechanisms, thereby improving performance across various benchmarks.

**Table 3:** The summary of baseline competencies in the experiments is presented as follows: a check mark ($\checkmark$) indicates the baseline’s ability to perform the sub-task, while a cross mark ($\times$) indicates the baseline’s inability to perform the sub-task.

Baselines	Basic module	Datasets	ATE	OTE	ALSC	AOE	AESC	AOP	ASTE
Li-Unified+	LSTM	D1,D2	$\checkmark$	$\checkmark$	$\checkmark$	X	$\checkmark$	$\checkmark$	$\checkmark$
OTE-MTL	Multi-task	D2	$\checkmark$	$\checkmark$	X	X	X	X	$\checkmark$
RINANTE+	LSTM+CRF	D1,D2	$\checkmark$	$\checkmark$	$\checkmark$	X	$\checkmark$	$\checkmark$	$\checkmark$
TS	LSTM+GCN	D1,D2	$\checkmark$	$\checkmark$	$\checkmark$	X	$\checkmark$	$\checkmark$	$\checkmark$
CMLA+	Attention	D1,D2	$\checkmark$	$\checkmark$	$\checkmark$	X	$\checkmark$	$\checkmark$	$\checkmark$
SPAN-ASTE	Bi-LSTM+BERT	D2	$\checkmark$	$\checkmark$	$\checkmark$	X	X	X	$\checkmark$
EMC-GCN	BERT	D1,D2	X	X	X	X	X	X	$\checkmark$
JET-BERT	BERT	D1,D2	$\checkmark$	$\checkmark$	$\checkmark$	X	$\checkmark$	$\checkmark$	$\checkmark$
DMRC	BERT	D1,D2	$\checkmark$	X	$\checkmark$	$\checkmark$	$\checkmark$	$\checkmark$	$\checkmark$
BMRC	BERT	D2	$\checkmark$	$\checkmark$	$\checkmark$	$\checkmark$	$\checkmark$	$\checkmark$	$\checkmark$
SentiPrompt	BART	D1,D2	X	X	X	X	$\checkmark$	$\checkmark$	$\checkmark$
BART-ABSA	BART	D1,D2	$\checkmark$	$\checkmark$	$\checkmark$	$\checkmark$	$\checkmark$	$\checkmark$	$\checkmark$
SE-GCN	BERT	D1,D2	$\checkmark$	$\checkmark$	$\checkmark$	$\checkmark$	$\checkmark$	$\checkmark$	$\checkmark$
Our	BERT	D1,D2	$\checkmark$	$\checkmark$	$\checkmark$	$\checkmark$	$\checkmark$	$\checkmark$	$\checkmark$

**Table 4:** Empirical outcomes for the tasks of Opinion Target Extraction (OTE), Aspect-Based Sentiment Classification (AESC), Aspect and Opinion Pairing (AOP), and Aspect Sentiment Triplet Extraction (ASTE) on the dataset D1.

Model	OTE			AESC			AOP			ASTE		
	P	R	F1	P	R	F1	P	R	F1	P	R	F1
Li-Unified+	81.20	83.18	82.13	73.15	74.44	73.79	44.37	73.67	55.34	41.44	68.79	51.68
RINANTE+	81.06	72.05	76.29	48.97	47.36	48.15	42.32	51.08	46.29	31.07	37.63	34.03
TS	84.72	80.39	82.45	76.60	67.84	71.95	47.76	68.10	56.10	44.18	62.99	51.89
OTE-MTL	–	–	–	–	–	–	–	–	–	66.04	56.25	60.62
RACL-BERT	82.28	90.49	**86.19**	75.57	82.23	78.76	73.58	67.87	70.61	62.64	57.77	60.11
SE-GCN	86.02	85.29	85.65	82.52	77.04	79.68	78.92	78.75	**78.83**	72.51	75.29	73.87
EMC-GCN	–	–	–	–	–	–	–	–	–	71.21	72.39	71.78
BART-ABSA	–	–	–	–	78.47	–	–	77.68	–	–	72.46	
SentiPrompt	–	–	–	–	–	–	–	–	–	72.79	72.94	72.86
DMRC	–	–	–	76.84	76.31	76.57	76.23	73.67	74.93	71.55	69.14	70.32
BMRC	87.22	82.90	84.99	77.74	75.10	76.39	76.91	75.59	76.23	71.32	70.09	70.69
SynGen	–	–	–	–	–	79.72	–	–	77.59	–	–	**74.02**
Ours	85.71	86.19	85.95	82.52	77.60	**79.94**	78.82	78.75	78.82	72.51	75.29	73.85
*Results for OTE, AESC, AOP, and ASTE on Res14.*												
Li-Unified+	79.18	75.88	77.44	64.95	64.95	64.95	52.75	61.75	56.85	43.34	50.73	46.69
RINANTE+	77.40	57.00	65.70	46.20	37.40	41.30	37.10	33.90	35.40	29.40	26.90	28.00
TS	78.07	78.07	78.02	67.65	64.02	65.79	49.22	65.70	56.23	40.97	54.68	46.79
OTE-MTL	–	–	–	–	–	–	–	–	–	57.51	43.96	49.76
RACL-BERT	76.25	83.96	79.91	68.35	70.72	69.51	67.89	63.74	65.46	55.45	52.53	53.95
SE-GCN	81.32	79.41	80.10	69.70	70.22	69.96	74.75	65.71	69.94	65.09	60.66	62.80
EMC-GCN	–	–	–	–	–	–	–	–	–	61.54	62.47	61.93
BART-ABSA	–	–	–	–	–	69.95	–	–	67.98	–	–	60.11
SentiPrompt	–	–	–	–	–	–	–	–	–	62.97	62.06	62.51
DMRC	–	–	–	66.84	63.52	65.14	72.43	58.90	64.97	63.78	51.87	57.21
BMRC	82.99	73.23	77.79	72.41	62.63	67.16	71.59	65.89	68.60	63.71	58.63	61.05
SynGen	–	–	–	–	–	**71.61**	–	–	69.35	–	–	**64.06**
Ours	81.64	79.83	**80.70**	70.82	69.42	70.11	74.51	66.4	**70.24**	65.49	59.43	62.33

*Results for OTE, AESC, AOP, and ASTE on Res15.*												
Li-Unified+	76.62	74.90	75.70	66.28	60.71	63.38	52.29	52.94	52.56	42.25	42.78	42.47
RINANTE+	78.20	62.70	69.60	41.20	33.20	36.70	34.40	26.20	29.70	23.10	17.60	20.00
TS	78.22	71.84	74.84	63.15	61.55	62.34	50.00	58.47	53.85	40.40	47.24	43.50
OTE-MTL	–	–	–	–	–	–	–	–	–	50.52	39.71	44.31
RACL-BERT	77.58	81.22	79.36	59.75	68.90	64.00	54.22	66.94	59.90	41.99	51.84	46.39
SE-GCN	81.78	74.89	80.26	68.09	68.89	68.48	69.65	66.53	68.06	57.17	64.83	60.76
EMC-GCN	–	–	–	–	–	–	–	–	–	61.70	56.26	58.81
BART-ABSA	–	–	–	–	–	68.17	–	–	66.11	–	–	57.59
SentiPrompt	–	–	–	–	–	–	–	–	–	63.40	58.60	60.90
DMRC	–	–	–	67.45	61.96	64.59	65.43	61.43	63.37	57.39	53.88	55.58
BMRC	84.67	67.18	74.90	72.73	62.59	67.27	74.11	61.92	67.45	65.12	54.41	59.27
SynGen	–	–	–	–	–	**70.06**	-	-	68.53	-	-	60.71
Ours	82.32	79.41	**80.78**	70.20	69.22	69.74	74.75	65.71	**69.96**	64.09	59.6	**61.72**
*Results for OTE, AESC, AOP, and ASTE on Lap14.*												
Li-Unified+	79.84	86.88	83.16	66.33	74.55	70.20	46.11	64.55	53.75	38.19	53.47	44.51
RINANTE+	75.0	42.4	54.1	49.4	36.7	42.1	35.7	27.0	30.7	27.1	20.5	23.3
TS	81.09	86.67	83.73	71.18	72.3	71.73	52.35	70.5	60.04	46.76	62.97	53.62
OTE-MTL	–	–	–	–	–	–	–	–	–	–	–	–
RACL-BERT	82.52	91.4	**86.73**	68.53	78.52	73.19	72.77	71.83	72.29	60.78	60.0	60.39
SE-GCN	83.87	87.39	85.59	77.98	74.32	76.10	79.38	77.0	**78.16**	71.84	69.67	70.74
EMC-GCN	–	–	–	–	–	–	–	–	–	65.62	71.30	68.33
BART-ABSA	–	–	–	–	–	75.69	–	–	77.38	–	–	69.98
SentiPrompt	–	–	–	–	–	–	–	–	–	70.20	73.35	71.74
DMRC	–	–	–	69.18	72.59	70.84	77.06	74.41	75.71	68.6	66.24	67.4
BMRC	85.31	83.01	84.13	73.69	72.69	73.18	76.08	76.99	76.52	67.74	68.56	68.13
SynGen	–	–	–	–	–	77.51	–	–	77.34	–	–	71.26
Ours	84.34	88.09	86.16	78.23	77.98	**78.1**	80.25	76.05	78.11	73.16	71.20	**72.20**
*Results for OTE, AESC, AOP, and ASTE on Res16.*												

Bold values are the best-performing results.

### Performance evaluation and comparative analysis

Our experimental evaluation on the D1 dataset presented in Table 4 included a variety of models handling tasks such as OTE, AESC, AOP, and ASTE. These models were assessed on their precision, recall, and F1-score metrics, providing a comprehensive view of their performance in Aspect Based Sentiment Analysis.

The “Ours” model showcased consistent high performance across all tasks, especially notable in its F1-scores. This indicates a well-balanced approach to precision and recall, crucial for nuanced tasks in natural language processing. SE-GCN also emerged as a top performer, particularly excelling in F1-scores, which suggests its efficiency in dealing with the complex challenges of sentiment analysis.

In the specific task of OTE, models like SE-GCN, BMRC, and “Ours” achieved high F1-scores, indicating their effectiveness in accurately identifying opinion terms within texts. For AESC, “Ours” and SE-GCN performed exceptionally well, demonstrating their ability to effectively extract and analyze aspects and sentiments in tandem.

In the Aspect-Opinion Pairing task, “Ours” and SE-GCN showed remarkable proficiency, suggesting their adeptness at correctly pairing aspects with corresponding opinions. Additionally, in the ASTE task, our model demonstrated superior performance, underlining its capability in intricately extracting linked aspect-sentiment entities.

When comparing our model to traditional models like Li-Unified+ and RINANTE+, it is evident that “Ours” outperforms them in almost all metrics. This superiority could be attributed to more advanced or specialized methodologies employed in our model. RACL-BERT also showed significant performance in certain tasks, likely benefiting from the advanced contextual understanding provided by BERT embeddings. The TS model, while not topping any category, showed consistent performance across tasks, suggesting its robustness.

An interesting observation from the results is the trade-off between precision and recall in several models. This indicates potential areas for improvement in future research. The selection of a model for practical applications should consider specific needs, such as the importance of precision over recall or vice versa.

These results indicate that there is room for enhancement in the field, particularly in balancing precision and recall. Future research could explore integrating context-aware embeddings and sophisticated neural network architectures to enhance performance in Aspect Based Sentiment Analysis.

In conclusion, our model demonstrates excellent performance across various tasks in ABSA on the D1 dataset, suggesting its potential for comprehensive and nuanced sentiment analysis in natural language processing. However, the choice of the model for specific applications should be aligned with the unique requirements of the task, considering the inherent trade-offs in precision, recall, and the complexities of natural language understanding. This study opens avenues for further research to enhance the accuracy and effectiveness of sentiment analysis models.

**Table 5:** Empirical findings for aspect sentiment triplet extraction (ASTE) on the D2 dataset.

Model	Lap14			Res14			Res15			Res16		
	P	R	F1	P	R	F1	P	R	F1	P	R	F1
Li-Unified+	40.56	44.28	42.34	41.04	67.35	51.00	44.72	51.39	47.82	37.33	54.51	44.31
RINANTE+	21.71	18.66	20.07	31.42	39.38	34.95	29.88	30.06	29.97	25.68	22.30	23.87
TS	37.38	50.38	42.87	43.24	63.66	51.46	48.07	57.51	52.32	46.96	64.24	54.21
JET-BERT	55.39	47.33	51.04	70.56	55.94	62.40	64.45	51.96	57.53	70.42	58.37	63.83
SE-GCN	65.65	54.77	**59.72**	70.03	67.47	68.73	65.38	61.88	63.58	73.72	69.28	71.43
SentiPrompt	61.30	55.32	58.15	66.10	63.37	64.71	61.81	62.06	61.93	68.66	69.04	68.85
BART-ABSA	61.41	56.19	58.69	65.52	64.99	65.25	59.14	59.38	59.26	66.60	68.68	67.62
SPAN-ASTE	63.44	55.84	59.38	72.89	70.89	71.85	62.18	64.45	63.27	69.45	71.17	70.26
Ours	66.35	54.09	59.59	70.60	67.70	**69.15**	65.38	62.98	**64.02**	73.17	70.08	**71.49**

Bold values are the best-performing results.

In Table 5, we observe a detailed comparison of various models for ASTE across four datasets: Lap14, Res14, Res16, and Res15. The evaluation metrics-Precision (P), Recall (R), and F1-score (F1)-provide a comprehensive view of each model’s performance in complex sentiment analysis tasks. Notably, SE-GCN stands out in the Lap14 dataset, achieving the highest F1-score (59.72), which reflects its effective handling of sentiment relationships. However, our model demonstrates exceptional consistency across all datasets, either closely matching or surpassing SE-GCN in terms of F1-scores. This is particularly evident in the Res14 and Res15 datasets, where our model records the highest F1-scores, showcasing its precision and robustness in sentiment analysis.

While other models like SPAN-ASTE and BART-ABSA show competitive performances, they are slightly outperformed by the leading models. In the Res16 dataset, our model continues its dominance with the highest F1-score (71.49), further establishing its efficacy in ASTE tasks. This performance indicates a refined balance in identifying and linking aspects and sentiments, a critical aspect of effective sentiment analysis. In contrast, models such as RINANTE+ and TS, despite their contributions, show room for improvement, especially in achieving a better balance between precision and recall.

The results presented in Table 5 emphasize the varying efficacy of models across different datasets. Each dataset’s unique characteristics, including the complexity of language and the nature of expressed aspects and sentiments, significantly impact model performance. The consistent top-tier performance of our model across diverse datasets highlights its adaptability and nuanced understanding of sentiment dynamics. Such adaptability is crucial in real-world scenarios, where data variability is a common challenge. Overall, these findings from Table 5 underscore the significance of developing versatile and robust models for Aspect Based Sentiment Analysis, capable of adeptly handling a variety of linguistic and contextual complexities.

## Model analysis

### Ablation study

The ablation study results reveal several important insights about the contributions of various components to the performance of our model. Firstly, it is evident that the complete model configuration comprising refinement processes, syntactic features, and the integration of the MLEGCN and attention modules-consistently yields the highest F1 scores across both the Res14 and Lap14 datasets. This underscores the synergy between the components, suggesting that each plays a crucial role in the model’s ability to effectively process and analyze linguistic data. Particularly, the removal of the refinement process results in a uniform decrease in performance across all model variations and datasets, albeit relatively slight. This suggests that while the refinement process significantly enhances the model’s accuracy, its contribution is subtle, enhancing the final stages of the model’s predictions by refining and fine-tuning the representations.

**Table 6:** F1 score for abalation study.

	Our Model		Without Refinement		Without Syntatic Features		Without MLEGCN & Attention	
Model	Res14	Lap14	Res14	Lap14	Res14	Lap14	Res14	Lap14
OTE	85.95	80.78	85.04	79.02	85.44	80.15	84.65	79.71
ATE	85.95	82.62	84.10	81.83	85.05	81.89	83.69	79.42
AOE	78.72	69.94	76.82	68.07	78.02	69.01	76.98	67.22
ALSC	81.95	78.25	79.98	76.83	81.09	77.45	78.93	74.48
AESC	79.94	69.74	78.52	68.87	79.83	68.50	76.67	65.89
AOPE	78.82	70.24	76.94	68.54	77.97	69.00	76.02	66.88
ASTE	73.85	61.72	71.81	59.65	73.02	60.70	70.94	59.00

Table 6 More pronounced are the effects observed from the removal of syntactic features and the MLEGCN and attention mechanisms. The exclusion of syntactic features leads to varied impacts on performance, with more significant declines noted in tasks that likely require a deeper understanding of linguistic structures, such as AESC, AOPE, and ASTE. This indicates that syntactic features are integral to the model’s ability to parse complex syntactic relationships effectively. Even more critical appears the role of the MLEGCN and attention mechanisms, whose removal results in the most substantial decreases in F1 scores across nearly all tasks and both datasets. This substantial performance drop highlights their pivotal role in enhancing the model’s capacity to focus on and interpret intricate relational dynamics within the data. The attention mechanisms, in particular, are crucial for weighting the importance of different elements within the input data, suggesting that their ability to direct the model’s focus is essential for tasks requiring nuanced understanding and interpretation.

These observations from the ablation study not only validate the design choices made in constructing the model but also highlight areas for further refinement and exploration. The consistent performance degradation observed upon the removal of these components confirms their necessity and opens up avenues for further enhancing these aspects of the model. Future work could explore more sophisticated or varied attention mechanisms and delve deeper into optimizing syntactic feature extraction and integration to boost the model’s performance, particularly in tasks that heavily rely on these components.

### Syntactic features qualitative analysis

**Figure 6:**
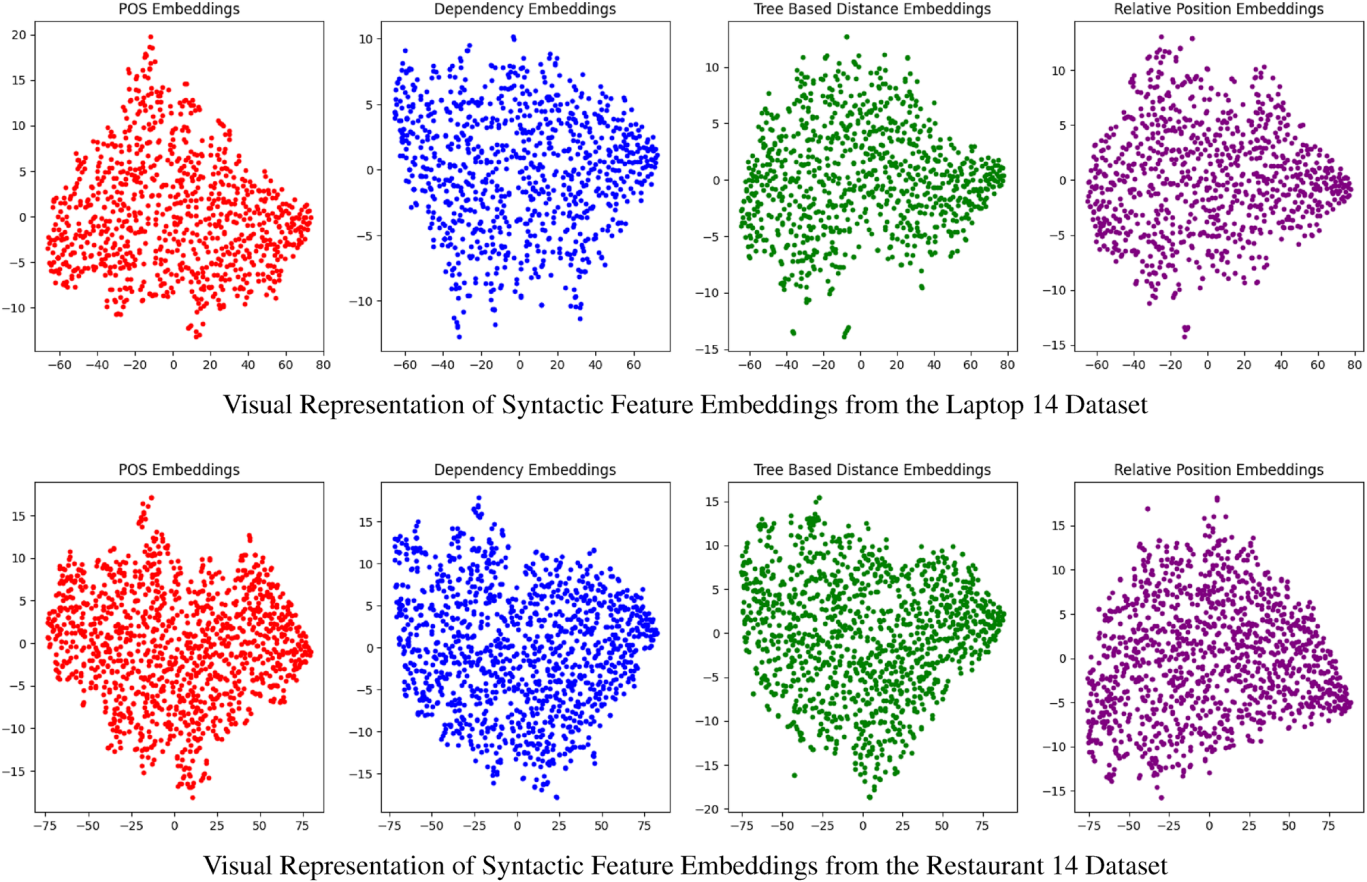
Comprehensive visualization of the embeddings for four key syntactic features.

These visualizations serve as a form of qualitative analysis for the model’s syntactic feature representation in Figure 6. The observable patterns in the embedding spaces provide insights into the model’s capacity to encode syntactic roles, dependencies, and relationships inherent in the linguistic data. For instance, the discernible clusters in the POS embeddings suggest that the model has learned distinct representations for different grammatical categories, which is crucial for tasks reliant on POS tagging. Moreover, the spread and arrangement of points in the dependency embeddings indicate the model’s ability to capture a variety of syntactic dependencies, a key aspect for parsing and related NLP tasks. Such qualitative observations complement our quantitative findings, together forming a comprehensive evaluation of the model’s performance.

### Case study

The presented case study offers a meticulous examination of our model’s capabilities in Aspect-Based Sentiment Analysis (ABSA) against established benchmarks such as BART-ABSA and BMRC presented in Table 7. Through a diverse array of product reviews, our model consistently demonstrates superior accuracy in deciphering complex aspect-sentiment relationships. For example, in Review 3, our model accurately captures the nuanced sentiment ’superb’ associated with ’noise cancellation’ and the negative sentiment ’short’ tied to ’battery life,’ aligning perfectly with the ground truth. This precision is attributed to our model’s advanced linguistic feature extraction and refined sentiment contextualization, which outperforms the competing models, particularly in cases where the sentiment is subtle or the aspect term is compound. Moreover, the case study underscores the models’ error patterns, where BART-ABSA occasionally falters in associating sentiments with the correct aspects, and BMRC sometimes misinterprets complex sentiment expressions. In contrast, our model exhibits a robust understanding of intricate linguistic cues, leading to its enhanced performance. These case study insights not only reaffirm our model’s adeptness at tackling the multifaceted nature of sentiment analysis but also highlight its potential to serve as a formidable tool in understanding and quantifying nuanced customer feedback across various product domains.

**Table 7:** Different Models outputs for given Examples. Wrong predictions are indicated by the marker $\times$.

Models	Reviews
*Review 1: “This phone’s display is crisp but drains the battery quickly.”*	
Ground Truth	[(display, crisp, POS), (battery, drains quickly, NEG)]
BART-ABSA	[(phone, crisp, POS) $\times$, (battery, quickly, NEG)]
BMRC	[(display, sharp, POS), (battery, drains, NEG)]
Ours	[(display, crisp, POS), (battery, drains quickly, NEG)]
*Review 2: “The laptop is lightweight yet feels sturdy and durable.”*	
Ground Truth	[(laptop, lightweight, POS), (laptop, sturdy, POS), (laptop, durable, POS)]
BART-ABSA	[(laptop, portable, POS), (durable, sturdy, NEG) $\times$]
BMRC	[(laptop, light, POS), (laptop, sturdy and durable, POS) $\times$]
Ours	[(laptop, lightweight, POS), (laptop, sturdy, POS), (laptop, durable, POS)]
*Review 3: “The headphones have superb noise cancellation, but the battery life is short.”*	
Ground Truth	[(noise cancellation, superb, POS), (battery life, short, NEG)]
BART-ABSA	[(noise cancellation, superb, POS), (battery, short, NEG) $\times$]
BMRC	[(cancellation, superb, POS)$\times$, (battery life, short, POS) $\times$]
Ours	[(noise cancellation, superb, POS), (battery life, short, NEG)]
*Review 4: “The software is user-friendly but has some annoying bugs.”*	
Ground Truth	[(software, user-friendly, POS), (bugs, annoying, NEG)]
BART-ABSA	[(software, friendly, POS) $\times$, (bugs, annoying, NEG) $\times$]
BMRC	[(software, user-friendly, NEG)$\times$, (software, bugs, NEG) $\times$]
Ours	[(software, user-friendly, POS), (bugs, annoying, NEG)]
*Example 5: “The camera takes sharp photos, yet the battery drains quickly.”*	
Ground Truth	[(camera, sharp photos, POS), (battery, drains quickly, NEG)]
BART-ABSA	[(camera, photos, POS)$\times$, (battery, drains, NEG) $\times$]
BMRC	[(camera, sharp photos, POS), (battery, drains quickly, NEG)]
Ours	[(camera, sharp photos, POS), (battery, drains quickly, NEG)]

## Conclusion

This research presents a pioneering framework for ABSA, significantly advancing the field. The model uniquely combines a biaffine attention mechanism with a MLEGCN, adeptly handling the complexities of syntactic and semantic structures in textual data. This approach allows for precise extraction and interpretation of aspects, opinions, and sentiments. The model’s proficiency in addressing all ABSA sub-tasks, including the challenging ASTE, is demonstrated through its integration of extensive linguistic features. The systematic refinement strategy further enhances its ability to align aspects with corresponding opinions, ensuring accurate sentiment analysis. Overall, this work sets a new standard in sentiment analysis, offering potential for various applications like market analysis and automated feedback systems. It paves the way for future research into combining linguistic insights with deep learning for more sophisticated language understanding.

## Data Availability

The datasets analyzed during the current study are available in the Wu et al and Xu et al repositories, https://github.com/NJUNLP/GTS, https://github.com/xuuuluuu/SemEval-Triplet-data/tree/master/ASTE-Data-V2-EMNLP2020.
